# ggroups: an R package for pedigree and genetic groups data

**DOI:** 10.1186/s41065-020-00124-2

**Published:** 2020-05-04

**Authors:** Mohammad Ali Nilforooshan, Luis Antonio Saavedra-Jiménez

**Affiliations:** 1 Livestock Improvement Corporation, Private Bag 3016, Hamilton, 3240 New Zealand; 2 Universidad Autónoma Chapingo, Departamento de Zootecnia, México-Texcoco, Chapingo, C. P. 56230 Mexico

**Keywords:** Pedigree, Relationship matrix, Inverse, Genetic groups, Dominance, R

## Abstract

**Background:**

R is a multi-platform statistical software and an object oriented programming language. The package archive network for R provides CRAN repository that features over 15,000 free open source packages, at the time of writing this article (https://cran.r-project.org/web/packages, accessed in October 2019). The package ggroups is introduced in this article. The purpose of this package is providing functions for checking and processing the pedigree, calculation of the additive genetic relationship matrix and its inverse, which are used to study the population structure and predicting the genetic merit of animals. Calculation of the dominance relationship matrix and its inverse are also covered. A concept in animal breeding is genetic groups, which is about the inequality of the average genetic merits for groups of unknown parents. The package provides functions for the calculation of the matrix of genetic group contributions (**Q**). Calculating **Q** is computationally demanding, and depending on the size of the pedigree and the number of genetic groups, it might not be feasible using personal computers. Therefore, a computationally optimised function and its parallel processing alternative are provided in the package.

**Results:**

Using sample data, outputs from different functions of the package were presented to illustrate a real experience of working with the package.

**Conclusions:**

The presented R package is a free and open source tool mainly for quantitative geneticists and ecologists, who deal with pedigree data. It provides numerous functions for handling pedigree data, and calculating various pedigree-based matrices. Some of the functions are computationally optimised for large-scale data.

## Background

Pedigree information provides the principles of the traditional as well as modern animal breeding today. It provides key information about inheritance, rate of kinship between relatives, heritability and segregation of the phenotypic variance to genetic and non-genetic components, inbreeding, effective population size, population structure, and mating patterns. Pedigree information fitted into the pedigree relationship matrix (**A**) is used in the best linear unbiased prediction (BLUP) animal models [1]. BLUP is a mixed model equation system involving fixed and random (including animal genetic) effects for the prediction of animals’ breeding values. With genomic information and genomic relationship matrices becoming available, **A** is still needed in recent genetic evaluation models, such as single-step genomic BLUP [2, 3] and single-step marker effect model [4]. The inverse of **A** is needed in BLUP, and matrix inversion is computationally challenging. Inverting a matrix has a cubic computational cost relative to the dimension of the matrix. Henderson [5] and Quaas [6] invented a method for indirect inversion of **A**, with a linear computational cost.

Another concept in the genetic evaluation of animals is genetic groups (also called phantom parent groups). Genetic groups are for taking into account for the fact that unknown parents belong to different groups with different averages of genetic merit, depending on the birth year and the genetic background of the unknown parents. The recommended grouping strategy [7] is based on the 4 pathways of selection (sire of sons, sire of daughters, dam of sons, and dam of daughters), and the birth year of the progeny of the unknown parent. Sufficient number of unknown parents are required in each group to make accurate inferences about the group effects. Therefore, subsequent years might be merged. For some species, grouping might involve the breed and regions within a country [7]. With international genetic trade of genetic matrials, country of origin should be considered in forming genetic groups (e.g., in dairy cattle populations open to foreign genetic materials).

The aim of this study is to introduce an R package (ggroups [8]) for pedigree processing, obtaining pedigree-based parameters and matrices (including additive and dominance relationship matrices and their inverses), the contribution of genetic groups to the genetic merit of animals, and its correspondence matrix (**Q**).

There are a few R packages that have functions similar to some of the functions in ggroups. However, these packages also provide different functionalities for different user needs. Therefore, the aim is not putting these R packages into comparison. Some packages are written in C or C++ to increase the speed. R package ggroups is written in R for better readability in a high-level language, which can be more helpful for educational purposes.

## Implementation and results

In this section, the implementation of ggroups functions is explained with examples. This R package is available on CRAN (https://cran.r-project.org) and can be installed in R, using the command install.packages(“ggroups”). The convention of package::function was used to address functions from other R packages. For presenting most of the functions, the example pedigree in Quaas [9] was used:


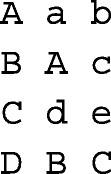


where, the columns correspond to animal, sire, and dam ID. Unknown parents a, b, c, d, and e were belonging to genetic groups, g1, g2, g2, g1, and g2, respectively. The results were presented to show a real experience of working with the package. Also, a pedigree of Mexican Braunvieh cattle (57,341 animals, 2,746 sires, 27,015 dams, 3,925 missing sires, and 3,258 missing dams) with 8 genetic groups was used for performance (runtime) testing. Where memory was a limitation (e.g., forming a 57,341 × 57,341 matrix), a subset of 3,000 animals (with 482 sires, 1,855 dams, 780 missing sires, and 704 missing dams) from this pedigree was used. Runtimes were measured on an octa-core Intel(R) Core(TM) i7-8650U with 16 Gb of RAM (Sys.i7-8650U.16).

### Pedigree renumbering

The first step of working with a pedigree involves converting alpha-numeric identities to numeric identities and order the pedigree. Most genetic evaluations softwares require a numerical pedigree, where numeric IDs need to be ascending from parent to progeny. Therefore, it is important that the progeny ID is greater than the parent ID. The main reason is that, to build **A** or **A**^−1^, animals should be ordered by parents preceding progeny. Function renum does renumbering and ordering the pedigree, so that progeny’s ID is greater than parent’s ID. Below, the example pedigree is used, with unknown parents replaced with the corresponding genetic groups:


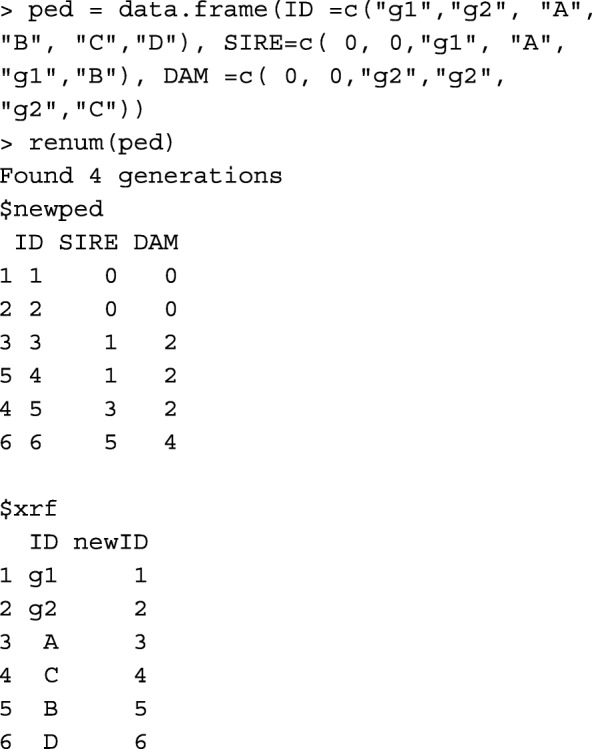


The output is a list of 2 data frames. The first is the renumbered pedigree, and the second is the cross-reference with columns corresponding to the original and renumbered ID. To get each data.frame separately, use renum(ped)$newped and renum(ped)$xrf.

Function orderPed from R package pedigree [10] (pedigree::orderPed) and AGHmatrix::datatreat [11] order the pedigree, but do not do renumbering. Applying pedigree::orderPed to the ped object above shows that ped does not need ordering.


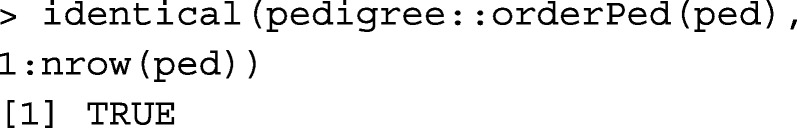


Function AGHmatrix::datatreat returns three lists for chronologically ordered individuals, their corresponding sires, and corresponding dams.


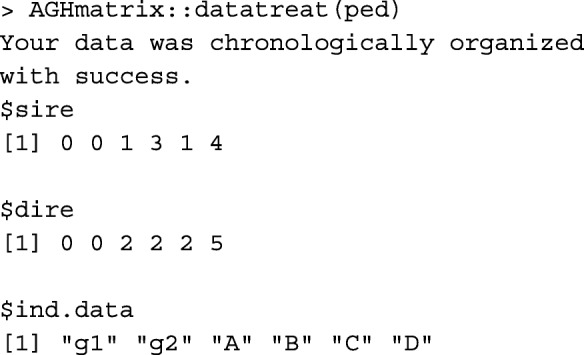


Loop(s) in the pedigree (an individual being ascendant to itself) would fail renum. Loops would appear among duplicated IDs in the pedigree with different parent information. If IDs are assigned so that parent ID is less than progeny ID, a duplicated row for the animal causing loop in the pedigree would show the opposite.

### Pedigree check

Function pedcheck performs basic checks on numeric pedigree. It is highly recommended to check the pedigree object with this function, before proceeding to other functions. The pedigree object is a data.frame with integer columns corresponding to the ID of animal, sire, and dam. Missing parents are set to 0. As an example, performing pedcheck on a faulty pedigree:


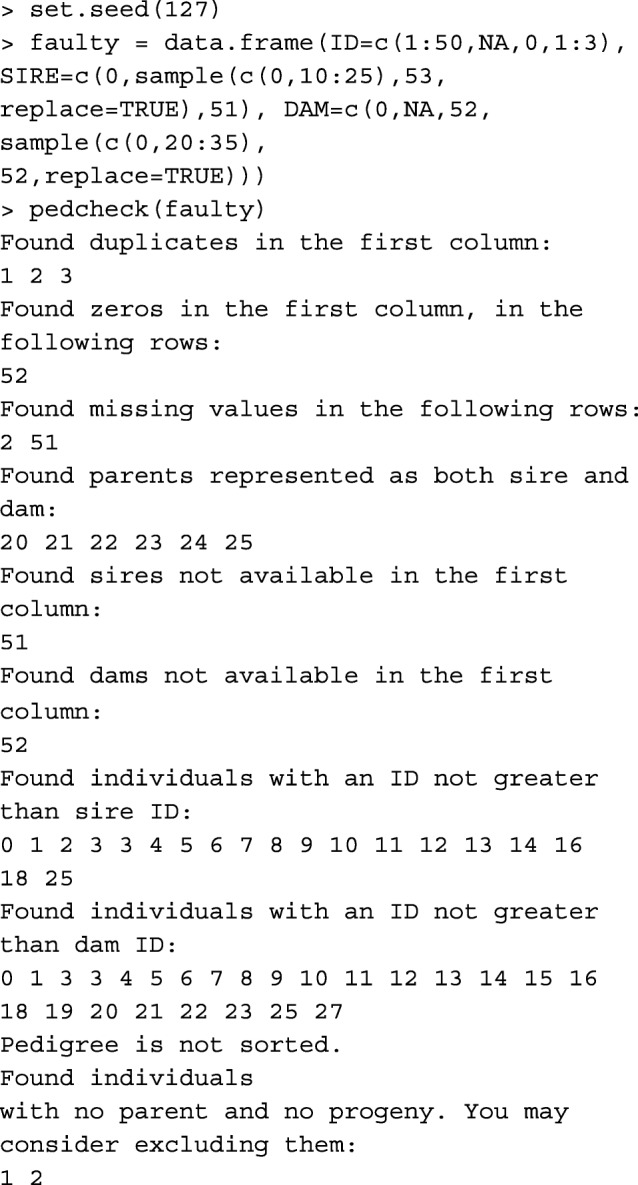


### All in and order the pedigree

Function gghead looks for possible parents missing in the first column of the pedigree, inserts them to the head of the pedigree, and orders the pedigree. Considering a pedigree with all missing parents replaced with the corresponding genetic groups, this functions appends the genetic groups to the head of the pedigree as the only IDs with missing parents. For example,


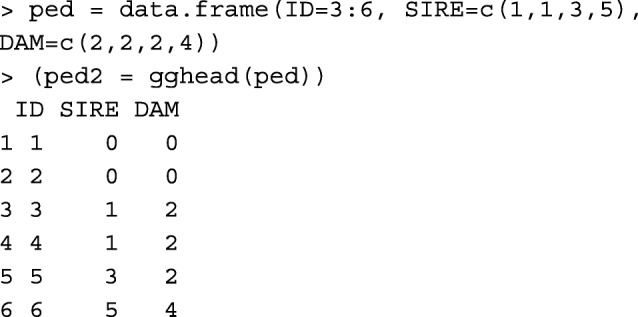


In comparison with R package pedigree [10], gghead function works similar to:


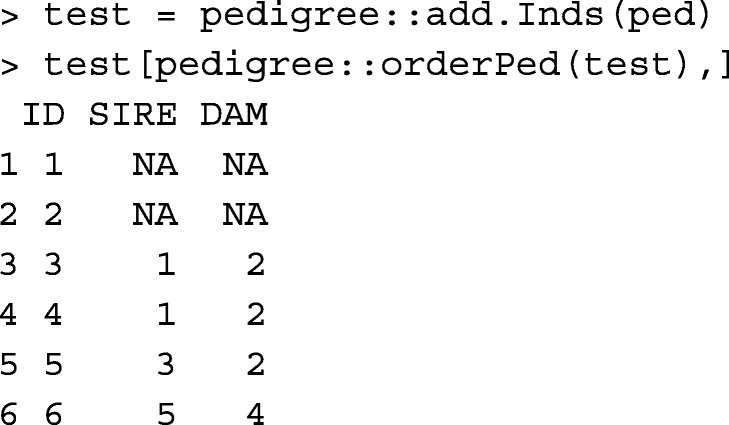


### Pedigree pruning

Pedigree pruning reduces memory usage and the time to reach convergence in the genetic evaluation. Usually, there are uninformative animals in the pedigree, which do not contribute or pass any information from descendants to ascendants. All those animals can be deleted from the pedigree for variance components estimation. In BLUP, some of those animals might be needed. Even though, they are uninformative, they might receive a predicted genetic merit from the information contributed by their informative relatives. Function prueped has two modes, strict and loose. The strict mode is recommended for variance components estimation, and the loose mode is recommended for BLUP. In the strict mode, animals without progeny and phenotype are deleted iteratively. Then, animals without known parents and progeny (if any) are deleted from the pedigree. In the loose mode, pedigree is upward extracted from phenotyped animals to their founders, and then pedigree is downward extracted from those founder animals. This less strict pruning leaves non-phenotyped animals with phenotyped relatives in the pedigree. Thus, they can receive a predicted genetic merit from BLUP. To test this function in both modes, consider animals 4, 5, and 6 are phenotyped, and animals 7 and 8 added to ped2:


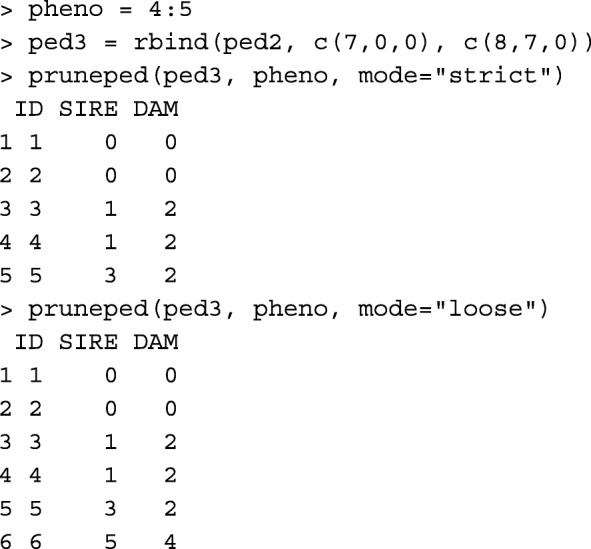


Function pedigree::trimPed [10] can be used to obtain the same output as function pruneped in the strict mode:


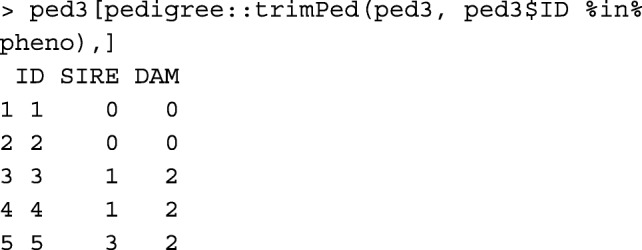


### Additive relationship matrix

Functions buildA and tabA create matrix **A** from the pedigree data.frame, in matrix and tabular-sparse formats, respectively. Function buildA is faster, but tabA is more memory-efficient (Table 1). Usually, relationship matrices and their inverses are saved in a tabular-sparse format. It reduces the memory usage and the size of the output file, as only the non-zero upper/lower triangular elements are saved. Considering the renumbered pedigree without genetic groups:

**Table 1:** Runtime ^*a*^ of different functions with two pedigree data

package::function	Time ^*b*^	Time ^*c*^
ggroups::tabA	–	13m:56s
pedigree::makeA	–	37s
ggroups::buildA	–	2s
pedigreemm::getA	–	1s
AGHmatrix::Amatrix	–	1s
pedigree::calcInbreeding	2s	1s
pedigreemm::inbreeding	1s	1s
ggroups::tabAinv	5m:28s	1s
pedigree::makeAinv	2s	1s
pedigreemm::getAInv	–	1s
ggroups::qmatL	–	2s
ggroups::qmatL	7m:27s	4s
ggroups::qmatXL	3m:31s	5s
nadiv::ggcontrib	–	1s
ggroups::tabD	–	39s
ggroups::buildD	–	2m
AGHmatrix::Amatrix^*d*^	–	1m:24s
ggroups::rg	41s ^*e*^	4s ^*e*^
ggroups::inb	35s ^*e*^	3s ^*e*^
ggroups::tabDinv	–	1m:29s
ggroups::tab2mat	–	31s ^*f*^
ggroups::mat2tab	–	4s ^*f*^

^a^Measured on an octa-core Intel(R) Core(TM) i7-8650U with 16 Gb of RAM, and runtimes shorter than 1s are presented as 1s

^b^A pedigree of 57,341 Mexican Braunvieh cattle (2,746 sires, 27,015 dams, and 8 genetic groups)

^c^A subset of 3,000 animals (482 sires, and 1,855 dams) from ^*b*^

^d^dominance=TRUE

^e^100 reiterations on random samples

^f^For the additive genetic relationship matrix


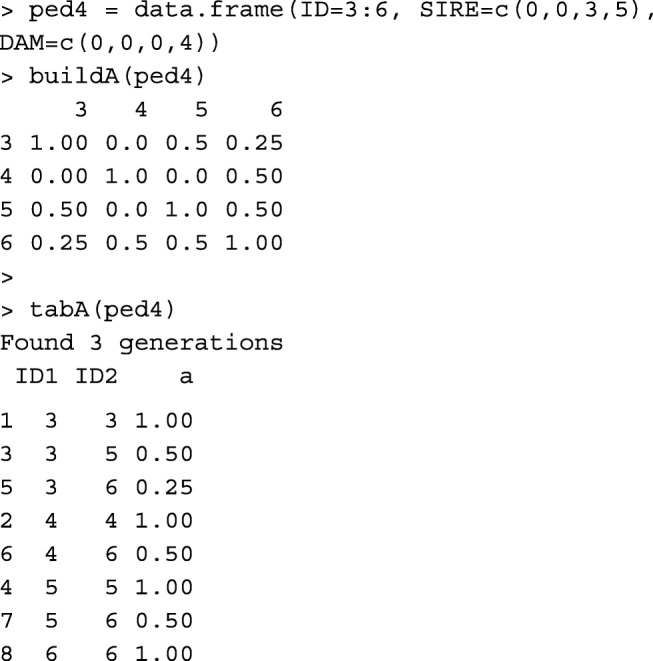


Functions pedigree::makeA [10], pedigreemm::getA [12] and AGHmatrix::Amatrix [11] also create matrix **A**.


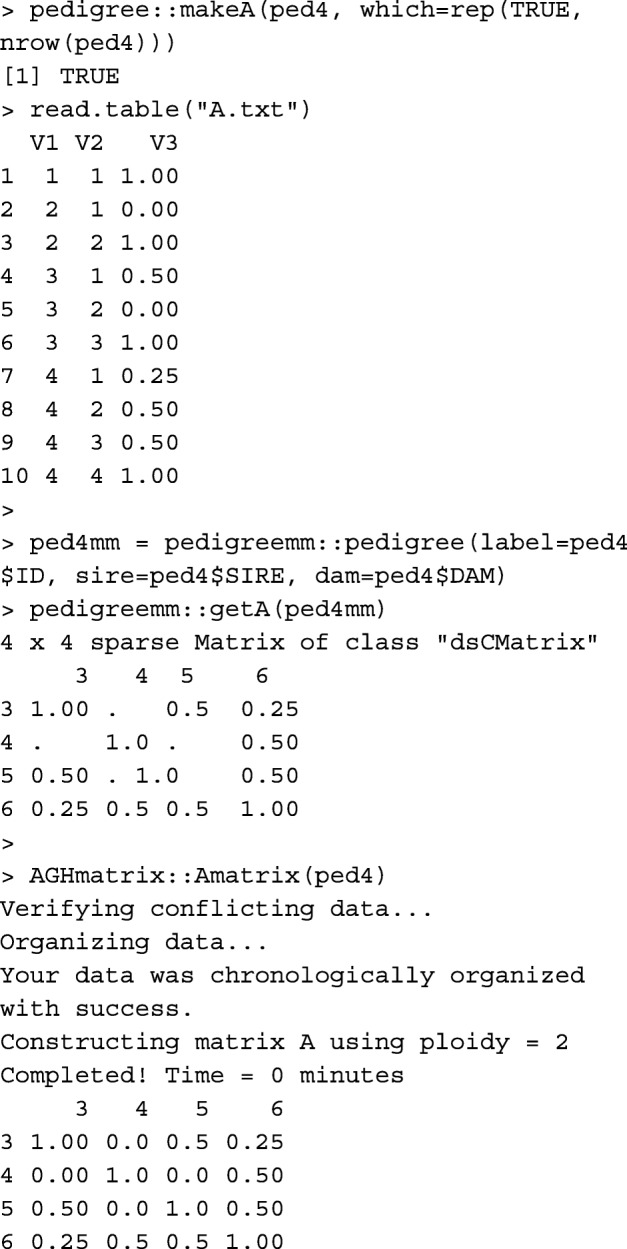


Please note that the first two columns in A.txt written by pedigree::makeA are the order of animals in the pedigree, not animal IDs. Table 1 provides runtime measures for buildA, tabA, pedigree::makeA, pedigreemm::getA, and AGHmatrix::Amatrix.

### Relationship coefficient

Whereas, functions buildA and tabA give relationship coefficients between all pairs of individuals, using these functions is computationally expensive (memory for buildA and runtime for tabA) if only the relationship coefficient between a pair of individuals is required, especially in a large pedigree. Instead, function rg can be used. For example, to get the relationship coefficient between individuals 3 and 6 in ped4, rg(ped4, 3, 6) results in: [1] 0.25. The same result can be obtained using function pedigree::makeA, but with less convenience.


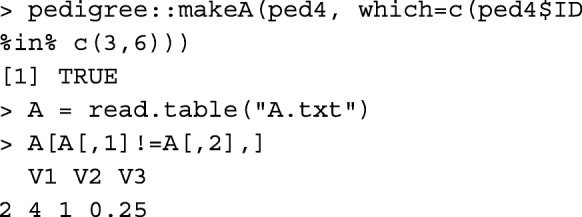


Calculating relationship coefficient between a pair of individuals using rg, information from their common parents are used only. Runtime of 100 reiterations of rg on random pairs of individuals is provided in Table 1.

### Inbreeding coefficient

If the inbreeding coefficient of an individual is of interest, using functions buildA and tabA is computationally expensive, especially in a large pedigree. Instead, function inb can be used. For example, to calculate the inbreeding coefficient of individual 6 in ped2, inb(ped2, 6) results in: [1] 0.25. Calculating inbreeding coefficient for an individual, information from parents’ common ancestors are used only. Runtime of 100 reiterations of inb on randomly chosen individuals is provided in Table 1.

Using package ggroups [8], there are 3 ways of obtaining inbreeding coefficients for all animals in the pedigree:


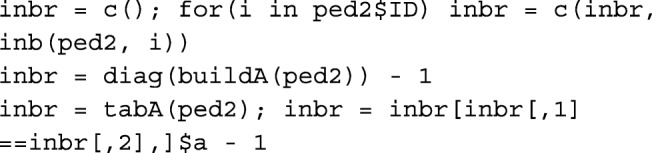


Functions pedigree::calcInbreeding [10] and pedigreemm::inbreeding [12] use fast and efficient algorithms [13] to compute inbreeding coefficients in large populations. Run time measures of pedigree::calcInbreeding and pedigreemm::inbreeding are provided in Table 1.

### Inverse of the additive relationship matrix

Function tabAinv builds **A**^−1^ in a tabular-sparse format. Compared to matrix data, handling tabular data takes a longer runtime. However, it is more memory-efficient for large pedigree. The pedigree object and inbreeding coefficients are required for tabAinv. None of the animals in ped4 were inbred. For example,


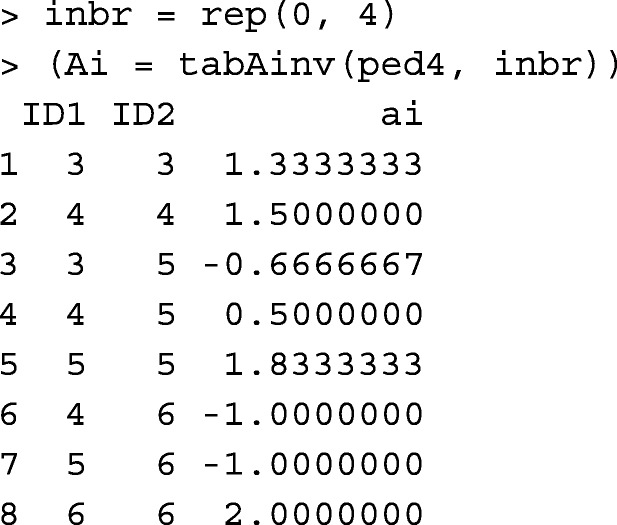


Function tabAinv performs similar to functions pedigree::makeAinv [10] and pedigreemm::getAInv [12].


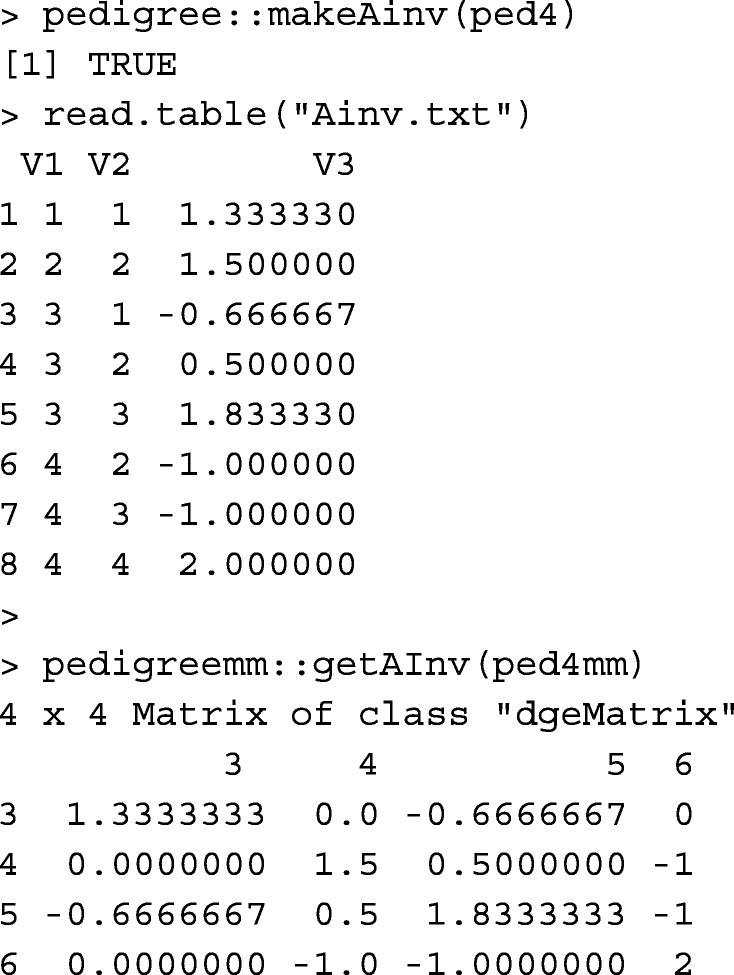


Please note that the first two columns in Ainv.txt written by pedigree::makeAinv are animal orders in the pedigree, not animal IDs. Table 1 provides runtime measures for tabAinv, pedigree::makeAinv, and pedigreemm::getAInv.

### Tabular to matrix and *vice versa*

Function tab2mat converts matrices from tabular-sparse format to matrix format. For example, **A**^−1^ created in the previous example is converted to matrix format:


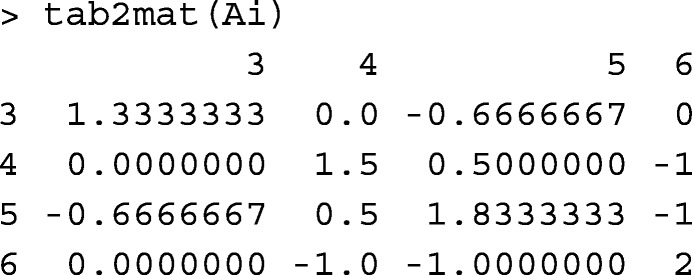


Function mat2tab converts matrices from matrix format to tabular-sparse format. For example, mat2tab(tab2mat(tabA(ped4))) returns tabA(ped4). Runtime records for converting a pedigree relationship matrix to tabular-sparse format (mat2tab) and *vice versa* (tab2mat) are provided in Table 1.

R package reshape2 [14] is a popular tool used for reshaping and transforming data. For example, it can be simply used for transforming a matrix to a tabular data.frame:


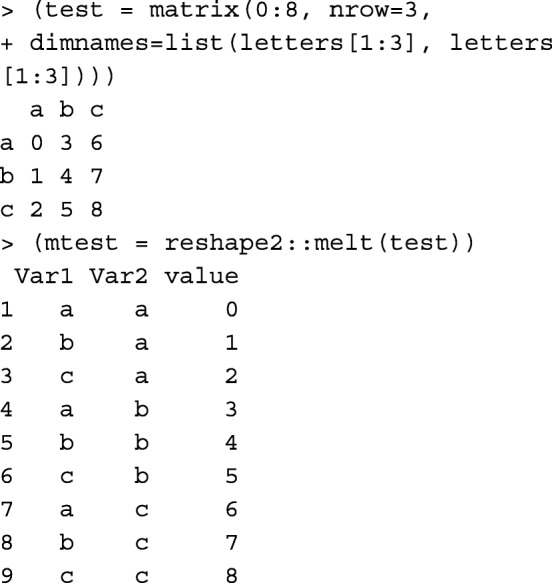


or to transform a tabular data.frame to a matrix:


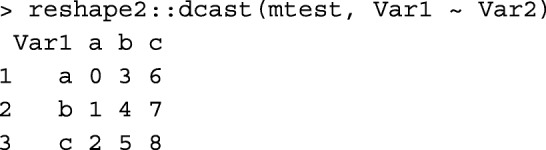


Unlike reshape2::melt and reshape2::dcast functions used above, mat2tab and tab2mat functions are specifically designed for symmetric matrices and tabular-sparse data, which reduces the number of rows in the data.frame to be read/written and kept in the memory.

### Genetic group contributions matrix

Function qmat creates matrix **Q**, which is the matrix of genetic group contributions to animals. According to Quaas [9], **Q**=(**I**−**P**)^−1^**P**_*b*_**Q**_*b*_, where (**I**−**P**)^−1^**P**_*b*_ is equal to the genetic relationship matrix between animals (rows) and unknown parents (columns), and **Q**_*b*_ is the incidence matrix of unknown parents (rows) and genetic groups (columns). Function qmatL is the computationally optimised version of qmat. It calculates each column of **Q** (each genetic group) separately, by calculating relationship coefficients between the genetic group of interest with animals that receive contribution from the genetic group (excluding missing parents, which receive full contribution).

The input pedigree should contain genetic groups (not unknown parents) as the only IDs with unknown parents, similar to the output from ped2 = gghead(ped). For example,


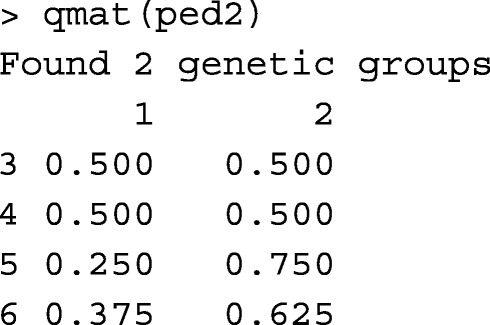


Function nadiv::ggcontrib [15] also returns matrix **Q** for pedigree and genetic groups. Using the Mexican Braunvieh pedigree on Sys.i7-8650U.16, both qmat and nadiv::ggcontrib aborted with "Error: cannot allocate vector of size 25.6 Gb" message. Function qmatL could even return **Q** on a machine with 4 Gb RAM (not all of it available). Functions qmat and nadiv::ggcontrib were tested on a server with 526 Gb of RAM. Function qmat returned **Q** after 17m:51s, and function nadiv::ggcontrib aborted with the following message:


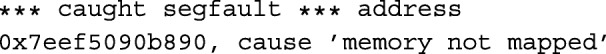


With the pedigree subset of 3,000 animals, function nadiv::ggcontrib successfully returned matrix **Q** on Sys.i7-8650U.16. Runtimes of qmat, qmatL, and nadiv::ggcontrib are provided in Table 1.

### Parallel computation of genetic group contributions matrix

In order to further speed up the calculation of **Q**, the process can take advantage of multicore processors and parallel computing. Function qmatXL is the parallelised version of qmatL. It requires a pedigree object as described for ped2, and the number of user-defined computational nodes. If the number of user-defined nodes was greater than the number of genetic groups, the number of genetic groups is considered for the number of nodes. This function requires R packages doParallel [16] and foreach [17], otherwise, an on-screen message will notify the user to install doParallel. Package foreach is a dependency for package doParallel. Thus, installing doParallel with its dependencies (i.e., install.packages(~doParallel~, dependencies=TRUE)) would automatically install foreach. The command for testing qmatXL with ped2 and 2 nodes is: qmatXL(ped2, 2).

Table 1 provides runtime records for qmatXL. The benefit from parallel processing is expected to increase with the greater number of genetic groups and computational nodes. For small pedigree, qmat and qmatL are fast enough that parallel processing from qmatXL shows no benefit (Table 1). This might be due to a small parallel processing overhead.

### Summing genetic merits and genetic group contributions

There are two main alternatives of introducing genetic groups in BLUP [9]:

1 $$ {\left[\begin{array}{ccc} \mathbf X' \mathbf R^{-1} \mathbf X &\mathbf X' \mathbf R^{-1} \mathbf Z &\mathbf X' \mathbf R^{-1} \mathbf{ZQ} \\ \mathbf Z' \mathbf R^{-1} \mathbf X &\mathbf Z' \mathbf R^{-1} \mathbf Z + \mathbf G^{-1} &\mathbf Z' \mathbf R^{-1} \mathbf{ZQ} \\ \mathbf Q' \mathbf Z' \mathbf R^{-1} \mathbf X &\mathbf Q' \mathbf Z' \mathbf R^{-1} \mathbf Z &\mathbf Q' \mathbf Z' \mathbf R^{-1} \mathbf{ZQ} \end{array}\right] \left[\begin{array}{c} \hat{\mathbf b} \\ \hat{\mathbf u} \\ \hat{\mathbf g} \end{array}\right] = \left[\begin{array}{ccc} \mathbf X' \mathbf R^{-1} \mathbf y \\ \mathbf Z' \mathbf R^{-1} \mathbf y \\ \mathbf Q' \mathbf Z' \mathbf R^{-1} \mathbf y \end{array}\right],}  $$

where $\hat {\mathbf b}, \hat {\mathbf u}$ and $\hat {\mathbf g}$ are the vectors for the predictions of fixed, genetic merit, and genetic group effects, respectively, with the corresponding matrices **X**, **Z**, and **ZQ**. Matrix **R**, and vector **y** correspond to the residuals and phenotypes. Using Quaas and Pollak [18] transformation, the equation system is transformed to:

2 $$ {\left[\begin{array}{ccc} \mathbf X' \mathbf R^{-1} \mathbf X &\mathbf X' \mathbf R^{-1} \mathbf Z &\mathbf 0 \\ \mathbf Z' \mathbf R^{-1} \mathbf X &\mathbf Z' \mathbf R^{-1} \mathbf Z + \mathbf G^{-1} &-\mathbf G^{-1} \mathbf Q \\ \mathbf 0 &-\mathbf Q' \mathbf G^{-1} &\mathbf Q' \mathbf G^{-1} \mathbf Q \end{array}\right] \left[\begin{array}{c} \hat{\mathbf b} \\ \mathbf Q \hat{\mathbf g} + \hat{\mathbf u} \\ \hat{\mathbf g} \end{array}\right] = \left[\begin{array}{ccc} \mathbf X' \mathbf R^{-1} \mathbf y \\ \mathbf Z' \mathbf R^{-1} \mathbf y \\ \mathbf 0 \end{array}\right].}  $$

If the genetic evaluation software applies Eq. (1) (e.g., MTDFREML [19]), function Qgpu can be used to obtain $\mathbf Q \hat {\mathbf g} + \hat {\mathbf u}$. Function Qgpu requires 2 arguments, the **Q** matrix and a data.frame with 2 columns for IDs and solutions [$\hat {\mathbf g}, \hat {\mathbf u}$], respectively. The order of solutions must be the order of columns and then the order of rows in **Q**. For example,


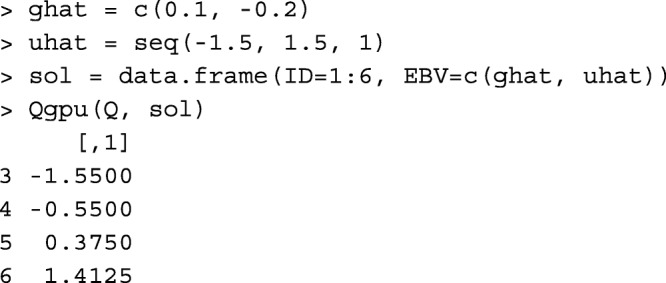


### Dominance relationship matrix

Whereas, additive genetic effects are inherited directly through individuals, inheritance of dominance effects are through pairs of mating individuals [20]. Hoeschele and VanRaden [20] defined pedigree-based dominance relationships by partitioning dominance effects into sire × dam subclass effects and within subclass deviations from within subclass average of dominance effects.

Functions buildD and tabD create matrix **D** from the pedigree data.frame, in matrix and tabular-sparse formats, respectively. Calculations are done for animals with both parents known. Other animals would only receive a diagonal value of 1. Below, functions buildD and tabD are shown in practice.


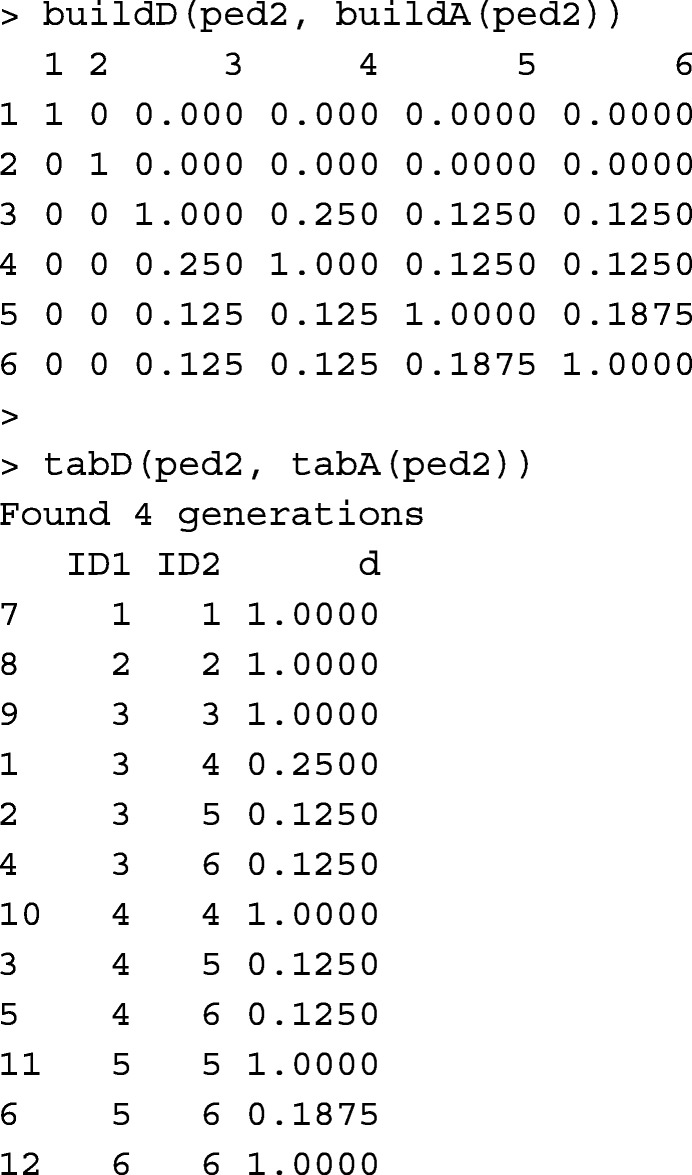


Function AGHmatrix::Amatrix [11] has a functionality similar to buildD.


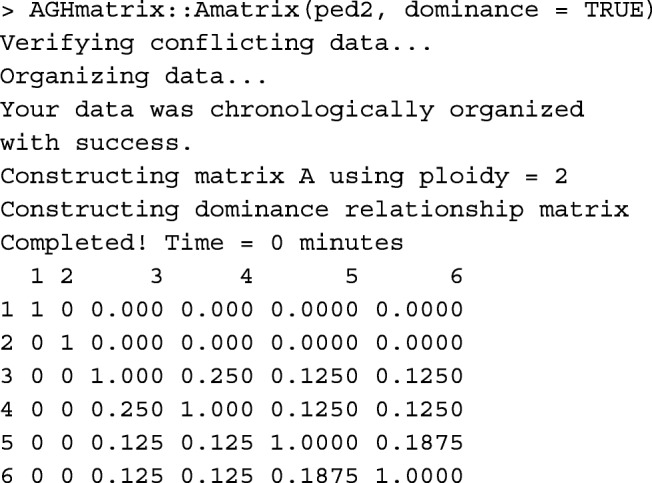


Runtime records of buildD, tabD, and AGHmatrix::Amatrix(dominance=TRUE) are provided in Table 1.

### Inverse of the dominance relationship matrix

Function tabDinv is used for obtaining **D**^−1^ in a tabular-sparse format from a pedigree object. For example,


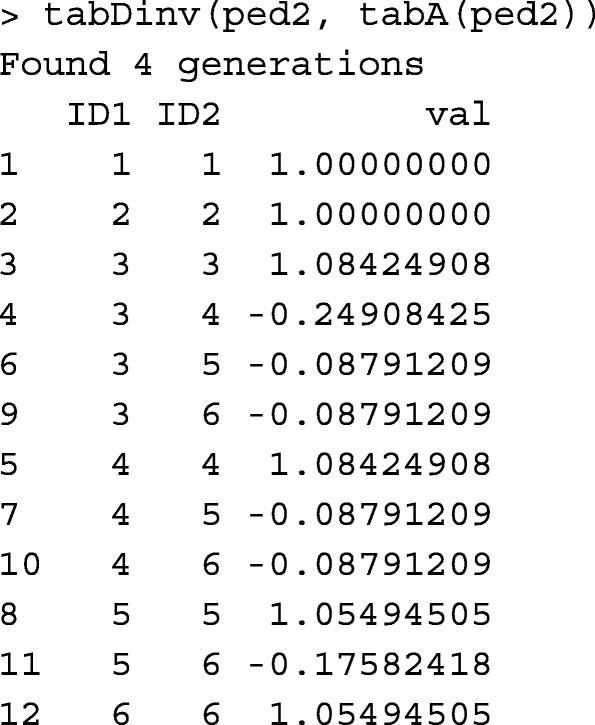


Runtime record of tabDinv is provided in Table 1.

### Other functions

There are other functions included in package ggroups, and possibly more functions will be added in the future. Some of the existing functions are offspring, pedup, and peddown. Function offspring reports the number of descendants from an individual, in each generation following that individual. Providing a list of animals such as the list of phenotyped or genotyped animals, the number of descendants from that list per generation is also provided. Function pedup extracts the pedigree for one or a group of individuals, by tracing their ancestors, for a defined or maximum possible number of generations. Similarly, function peddown extracts the pedigree for a group of individuals, by tracing their descendants down the pedigree.

## Conclusions

The R package ggroups is a useful, free and open source tool for animal breeders, researchers, and students, who work with pedigree data and R. It provides functions for the most important tasks related to working with pedigree, including creating the additive genetic relationship matrix and its inverse, and the matrix of genetic group contributions. The advantage of this package over other packages that also calculate the matrix of genetic group contributions, is that it has computationally optimised functions for large pedigree, which also can take advantage of multicore processors. In addition, R package ggroups provides other helpful functions such as functions for pedigree checking, renumbering and extraction, forming the dominance relationship matrix and its inverse, and converting tabular-sparse data to matrix, and *vice versa*. The results showed that functions dealing with data in matrix format are faster than functions dealing with data in tabular-sparse format. However, where memory of most personal computers fail forming large matrices for large pedigree, dealing with tabular-sparse data reduces the memory demand.

## Availability and requirements

- **Project name:** R package ggroups
- **Project home page:** https://cran.r-project.org/package=ggroups
- **Operating system(s):** Platform independent
- **Programming language:** R
- **Other requirements:**doParallel (≥1.0.14) and foreach (≥1.4.4) are required for function qmatXL.
- **License:** GPL-3

## Data Availability

https://cran.r-project.org/package=ggroups

## Abbreviations

BLUP: Best linear unbiased prediction
